# Caecal villi? A comparative histological and morphometric study of caecal and jejunal mucosa in adult rabbits

**DOI:** 10.1186/s13028-024-00770-w

**Published:** 2024-09-12

**Authors:** Adrian Florin Gal, Maria-Cătălina Matei-Lațiu, Călin Lațiu, Sanda Andrei, Vasile Rus

**Affiliations:** 1 Department of Cell Biology, Histology and Embryology, Faculty of Veterinary Medicine, University of Agricultural Sciences and Veterinary Medicine Cluj-Napoca, Manastur Street no 3- 5, Cluj-Napoca, 400372 Romania; 2 Department of Fundamental Sciences, Faculty of Animal Sciences and Biotechnologies, University of Agricultural Sciences and Veterinary Medicine Cluj-Napoca, Manastur Street no 3- 5, Cluj-Napoca, 400372 Romania; 3 Department of Biochemistry, Faculty of Veterinary Medicine, University of Agricultural Sciences and Veterinary Medicine Cluj-Napoca, Manastur Street no 3-5, Cluj-Napoca, 400372 Romania

**Keywords:** Caecum, Large intestine, Rabbit, Villi

## Abstract

**Background:**

Rabbits are herbivores with a distinctive digestive strategy that differs significantly from other caecal fermenters (e.g., horses, guinea pigs) and ruminants. In view of this, the current study aimed to highlight distinctive histological and morphometric features of the caecal mucosa in adult rabbits that accentuate its major role in digestion. The caecal and jejunal samples were harvested from five 1-year-old domestic rabbits and processed by regular paraffin-embedding histological technique followed by Goldner’s trichrome staining. A comprehensive morphological and morphometrical analysis of the jejunal mucosa vs. caecal mucosa was performed.

**Results:**

Microscopically, as in the case of the jejunal mucosa, the caecal mucosa presents long and often branched finger-like villi covered by a simple columnar epithelium mostly made of enterocytes with a prominent microvillous brush border. Besides, the caecal villi include a lacteal along with the villous muscle. Statistically, except for villus length, all the parameters assessed in the caecal mucosa, including villus width, villus count, thickness of the brush border and enterocyte/goblet cells ratio, revealed a high grade of similarities with the jejunal villi.

**Conclusions:**

According to the obtained results, the caecal mucosa in adult domestic rabbits includes unique features, namely caecal villi, structures infrequently presented in the large intestine of other adult mammals. Those structures once more emphasize the major role of the caecum not only in fermentation but also subliminally in local absorption. To our knowledge, this is the first reliable microanatomical and morphometric report of caecal villi in adult domestic rabbits.

**Supplementary Information:**

The online version contains supplementary material available at 10.1186/s13028-024-00770-w.

## Background

The order Lagomorpha includes two families, namely Leporidae (rabbits and hares) and Ochotonidae (pikas) [1–3]. Rabbits are herbivores with a distinctive digestive strategy, including physiology, that differs significantly from other caecal fermenters (e.g., horses, guinea pigs) and ruminants [1, 2]. The stomach usually contains roughly 15% of the ingesta present in the digestive tract, whereas the small intestine is short as compared to other species and includes approximately 12% of the total volume of the alimentary tract. As a comparison, the caecum usually contains approximately 40% of the ingesta of the gastrointestinal tract [3]. The colon includes two major segments, the cranial portion which is haustrated with prominent muscular taeniae, and includes multiple wart-like projections in the colonic mucosa that increase the surface area of absorption. The distal portion of the colon lacks haustra and taeniae [3].

In the caecum and colon of rabbits, a high retention of minute particles and solutes occurs due to a well-developed local mechanism, facilitating firstly an efficient fermentation of this portion of the diet, and, secondly, an unusually low digestibility of the crude fiber part of the diet. Consequently, a high feed intake is allowed despite the rapid digestive transit occurring, a fact that increases the total amount of extracted energy and minimizes the amount of stored fibers [1]. A separation of the digesta occurs in the colon due to the selective retention of small particles and fluid. In this sense, the regular peristalsis propels the larger, less dense fibers through the colon, whereas the small particles (i.e., the components with a higher density) and watery material retrograde to the caecum due to the contractions of the haustra of the colon. As a result, an extensive fermentation of the retained small particles and fluid happens in the caecum. Furthermore, the content of the caecum is expelled at times and consumed from the anus, a process called cecotrophy [2, 4]. As observed, the digestive tract in rabbits displays unique features, including the presence of a huge caecum designed for fermentation. Given this, rabbit microanatomy is the point of departure in understanding species-related physiology and pathology for such an essential and versatile species. Therefore, the current study presents distinctive histological and morphometric features of the caecal mucosa in adult rabbits that emphasize its major role in digestion.

## Methods

The tissue samples (i.e., jejunum and caecum) were collected from five healthy, 1-year-old male domestic rabbits (*Oryctolagus cuniculus domesticus* (Linnaeus, 1758)). The animals were housed under standard environmental conditions, in individual flat-deck wire cages (area 0.4 m^2^) with special bedding for rabbits (Cantacuzino Institute, Bucharest, Romania). The light, temperature and humidity of the room were kept constant during the housing period (14 h of light, 19 ± 1^o^C, and ~ 65% humidity). The physical activity was restricted to the cage size. The rabbits had ad libitum access to feed (commercial granular feed mixture – Cantacuzino Institute, Bucharest, Romania) and tap water. Experienced staff performed the care of the animals. The procedures were reduced to the minimum necessary to fulfil the purpose of the experiment. The use of the animals was approved by the Institutional Bioethics Committee (decision no. 289/390, 03.06.2023) and by the National Sanitary Veterinary and Food Safety Department (decision no. 384/20.08.2023). The samples harvested from the middle part of the jejunum and the body region of the non-folded area of the caecum underwent fixation in 10% buffered formalin and were successively embedded in paraffin. The tissue blocks were sectioned at 5 μm by using a Leica rotary microtome (model RM2125, Nussloch, Germany) and mounted on slides for staining by Goldner’s trichrome procedure [5]. After mounting, the stained slides were examined using an Olympus BX41 light microscope (Tokyo, Japan). The microscopic images were captured using an Olympus SC180 Microscope Digital Camera (Tokyo, Japan).

The morphometric assessment (villi length and width, thickness of the brush border, and enterocyte/goblet cells ratio) was reached using Olympus cellSens Imaging software. For all the measurements, the best-fit intercepted by the sections villi were selected. Accordingly, neither highly tortuous and incompletely sectioned villi, nor the ones out of the section plan, along with the ones that were not presenting the central axis were considered. Similarly, the villi that displayed a superimposed epithelium due to an oblique section, were avoided. The length and width of the intestinal villi were measured from 14 different villi, from the two intestinal segments. Since villi are not perfectly straight, the length was measured from its most apical region perpendicularly on the horizontal line at the villous base, just above the muscularis mucosae. Concerning the villi’s width dimension, three segments from each villus were assessed, i.e. the apical third (AT), middle third (MT), and basal third (BT). The width value for each villus was expressed as the mean value of the three measurements.

Microvilli length was measured from a total of 22 villi (11/intestinal segment). The thickness of the microvillous brush border was measured from three segments of the villi, including the apical third, middle third, and basal third. The average thickness of the brush border per villus was calculated based on the values achieved on the three above-mentioned segments.

A ratio of the enterocytes to goblet cells of the covering epithelium was calculated in the jejunum and caecum. The two cell types were counted from a total number of 10 villi from each intestinal segment assessed, by counting the enterocytes and goblet cells on 100 μm basal membrane length.

The statistical analysis was performed using Microsoft Excel and GraphPad Prism 8.0.1. The data were analyzed and expressed as mean ± standard deviation (SD)), Shapiro–Wilk test (to test normal distribution) and unpaired t-test (to establish if there were statistically significant differences between the number of enterocytes & goblet cells or the dimensions of the villi & microvillous brush border from the two intestinal segments. Statistically significant values were considered *P* < 0.05.

## Results

**Fig. 1:**
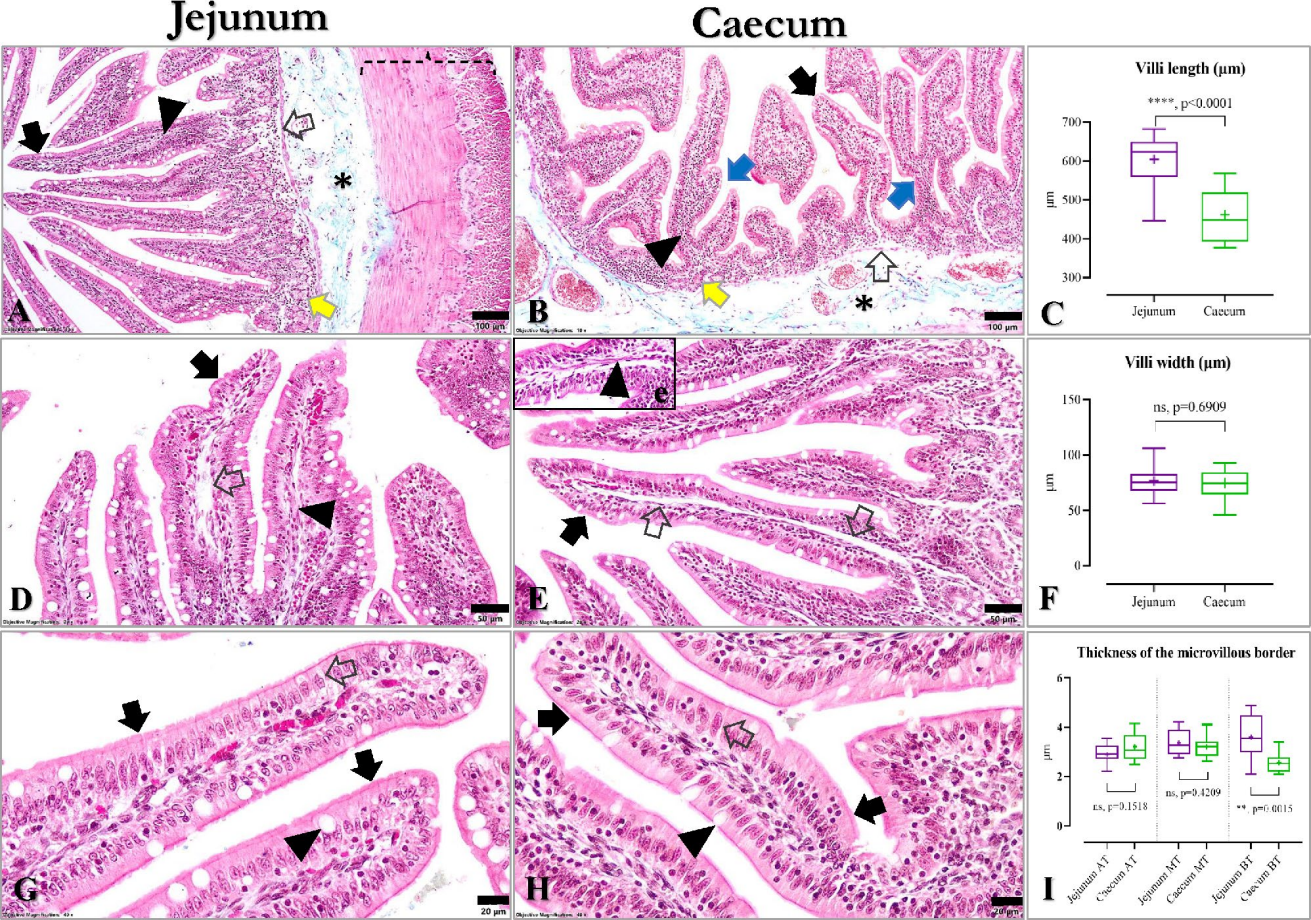
Comparative histological features of the jejunal and caecal mucosa in adult rabbits (Goldner’s trichrome stain). **A** Jejunum: long mucosal villi (black arrow) with associated lamina propria (arrowhead), which incorporates in its deeper zone jejunal glands (yellow arrow) flanked below by muscularis mucosae (empty arrow); the other jejunal layers include submucosa (asterisk), double layered muscularis externa (dashed accolade) covered externally by serosa. **B** Caecum: simple (black arrow) or branched (blue arrows) mucosal villi sustained by lamina propria (arrowhead), the last one presenting in the deeper zone the caecal glands (yellow arrow) flanked below by muscularis mucosae (empty arrow) and submucosa (asterisk) eventually. **C** Comparative morphological assessment of the length of jejunal and caecal villi (*p* < 0.0001). **D** Jejunum: the simple columnar covering epithelium of the villi made of enterocytes with brush border (black arrow), the central axis of villus containing the central chyliferous (empty arrow) and villous muscle (arrowhead). **E** Caecum: similar structures of a typical villus were identified in the caecal villi, including a simple columnar covering epithelium composed mostly of enterocytes with brush border (black arrow), the axially placed lacteal (empty arrows) along with villous muscle (e – arrowhead). **F** Comparative morphological assessment of the width of jejunal and caecal villi (*p* > 0.05). **G** Jejunum: a prominent brush border made of microvilli (black arrows) located on the apical poles of enterocytes (empty arrow), along with scattered goblet cells (arrowhead) can be observed. **H** Caecum: similarly to jejunal villi, a prominent microvillous brush border (black arrows) on the apical poles of enterocytes (empty arrow) and isolated goblet cells (arrowhead) were detected in the caecal mucosal villi. I - Comparative analysis of the microvilli-length in AT, MT and BT of jejunal and caecal villi (AT - *p* > 0.05; MT - *p* > 0.05; BT - *p* < 0.001)

Structurally, like the jejunum (Fig. 1A), the caecal wall includes the standard layers of a cavitary organ including mucosa (epithelium, lamina propria and muscularis mucosae), submucosa, muscularis externa, and serosa (Fig. 1B). Excepting the peculiarities recorded in the mucosa, no differences were identified in the structure of the other caecal layers. Concerning the latter, the caecal submucosa includes a highly vascularized loose connective tissue (Fig. 1B), muscularis externa consists of two planes that are inner circular and outer longitudinal smooth muscle layers. Concerning the caecal mucosa, some unusual features were identified compared to the mucosa of the large intestine overall in other mammals. Microscopically, the caecal mucosa presents long and often branched digitiform buds that closely mimic the villi (Fig. 1B). As in the case of jejunal villi (Fig. 1D, G), the caecal villi are covered by a simple columnar epithelium mostly made of enterocytes, which display a prominent brush border of microvilli (Fig. 1E, H). Internally, the caecal villi present a prominent lamina propria (Fig. 1B) that may include lymphoid tissue belonging to Gut-Associated Lymphoid Tissue (GALT). On the central longitudinal axis of the caecal villi, a prominent lymphatic vessel resembling the central chyliferous (lacteal; Fig. 1E) of the villi of the small intestine can be observed along with the villous muscle (myocytic villi; Fig. 1e). At the base of the caecal villi, short intestinal glands (Lieberkühn glands), can be seen lined by a simple columnar glandular epithelium surrounding a discreet lumen (Fig. 1B). Finally, the double-layered muscularis mucosae (i.e., internal circular and external longitudinal layers) delineate the mucosa from the submucosa (Fig. 1B).

A comparison of the villi from the jejunum vs. caecal villi was performed and several similitudes were identified as observed in Table 1.

**Table 1:** Comparative structural features of the jejunal and caecal villi in adult domestic rabbits

	Jejunum	Caecum
Enterocytes	+++	+++
Goblet cells	+	+
Brush border (microvilli)	+++	+++
Central chyliferous	present	present
Villous muscle	present	present

+ - poorly represented in number, +++ highly represented in number

Morphometrically, several similarities were observed between the two analysed intestinal segments (Table 2; Fig. 1F, I). Concerning the length of the villi, it can be observed that the average dimension is different between the two intestinal segments (604.7 ± 66.96 μm in jejunum vs. 461.8 ± 70.33 μm in caecum). As noticed, in jejunum the villi are significantly longer than in caecum. Moreover, the coefficient of variation (CV%) is higher in the caecum (15.23%) than in the jejunum (11.07%), a fact that suggests that in the first, the villus length variability is slightly more pronounced. Besides, the unpaired t-test results show that the difference in villus length between the two intestinal segments is statistically significant (*P* < 0.0001) (Fig. 1C).

**Table 2:** Comparative morphometric features of the jejunal and caecal villi in adult domestic rabbits

	Jejunum			Caecum		
Villi length (µm)	604.7 ± 66.96			461.8 ± 70.33		
Villi width (µm)	76.53 ± 13.73			74.46 ± 13.40		
Villi count/1000 µm	7.795 ± 1.262			6.695 ± 1.536		
Enterocyte/goblet cells ratio	9.48:1			7.52:1		
Thickness of the microvillous border	Avg 3.29 ± 0.43	AT	2.90 ± 0.39	Avg 2.99 ± 0.33	AT	3.21 ± 0.55
		MT	3.37 ± 0.48		MT	3.20 ± 0.43
		BT	3.60 ± 0.84		BT	2.56 ± 0.39

Avg – average value/villus; AT – villous apical third; MT - villous middle third; BT - villous basal third

However, the average width of villi is slightly similar between the two assessed intestinal segments (76.53 ± 13.73 μm in jejunum vs. 74.46 ± 13.40 μm in caecum). The CV% results are similar in the two intestinal segments assessed (17.94% in jejunum vs. 18.00% in caecum) suggesting that the variability of villus width is insignificant. The results of the unpaired t-test show no statistically significant differences in villus width (*P* > 0.05), emphasizing the above-mentioned observations (Table 2; Fig. 1F).

A length comparison of the microvillous brush border was performed in the three regions of each villus (i.e., AT, MT, BT). The mean value of the thickness of the brush border is similar in the two intestinal segments (3.29 ± 0.43 μm in jejunum vs. 2.99 ± 0.33 μm in caecum; *P* > 0.05). However, when each villous segment is analysed separately, some differences can be observed. In the first two segments of the villi (i.e. AT and MT) from the jejunum and caecum, the length of microvilli is similar (*P* > 0.05), whereas in the basal segment (BT) the brush border is thicker in the jejunum (3.60 ± 0.84 μm) than in caecum (2.56 ± 0.39 μm), a difference that is statistically significant according to unpaired t-test (*P* = 0.0015) (Table 2; Fig. 1I).

Concerning the distribution of enterocyte (Ec) and goblet cells (Gc) per 100 μm villous basal membrane of the jejunal vs. caecal covering epithelium, some differences are identified between the two segments. In the jejunum, a ratio of 9.48 Ec to 1 Gc was achieved which corresponds to a 17.06 Ec/1.79 Gc fraction, whereas in the caecum a ratio of 7.52 Ec to 1 Gc was obtained that corresponds to a fraction of 14.51 Ec/1.92 Gc. However, concerning the number of Ec and Gc between the two intestinal segments assessed, no statistically significant results were obtained by unpaired t-test (*P* > 0.05) (Table 2; Fig. 1 supplementary files).

Finally, a comparative analysis of the number of mucosal villi per 1000 μm mucosal length in the jejunum and caecum was performed. The mean count of villi is similar in the two assessed segments (7.795 ± 1.262 vs. 6.695 ± 1.536), the differences not being statistically significant (*P* = 0.2510) for unpaired t-test.

## Discussion

Mammals have evolved various macro- and histo-morphological structures to acclimate to a wide range of nutritional peculiarities. Adult rabbits, in contrast to other herbivores with caecal fermentation, possess caecal villi that closely resemble structurally the villi from the small intestine [1]. In our report, several resemblances were identified between jejunal villi vs. caecal villi. Excepting the villi length and the thickness of microvillous brush border in the BT that were significantly longer in the jejunum than in the caecum, no significant differences were observed for the other parameters assessed in the caecal vs. jejunal villi, including villus width, the thickness of the microvillous brush border in AT and MT, the Ec/Gc ratio, and the villi count/1000 µm mucosal length. Furthermore, a similarity was observed in the histomorphology of the caecal villi as compared to the jejunal villi, including by identifying both the villous muscle and central lacteal. To our knowledge, this is the first comprehensive description of caecal villi in adult rabbits, such structures being seldomly described before in the large intestine in adult mammals.

The structure of the gastrointestinal tract (GI) is a solid indicator of the food habits of a species. In this sense, some unusual peculiarities have been described in the digestive tract of some species, e.g. the neo-tropical opossum (*Didelphis marsupialis insularis*), in which the caecal mucosa has plicae circulares but without villi [6]. The uncommon presence of villi in the large intestine (i.e., caecal villi) was mentioned in some old reports in pika (*Ochotona princeps*) and red tree mouse (*Phenacomys longicaudus*) [7]. In pika (*O. princeps*), the caecal villi are flattened structures, ellipsoidal in cross-section, wider at the base as compared to the apex (i.e., similar to the finger of a rubber glove) [7]. The average length of the caecal villi in pika was 3 mm in length (length range 1.5–7.1 mm) and an average villus width of 0.45 mm; longer villi (6–7 mm) were occasionally detected and exhibited a more slender appearance. As a comparison, the villi described in pika are shorter, wider, and flatter as compared to the ones of the red tree mouse. Histologically, more smooth muscular tissue and less lymphoid tissue were identified in pika’s villi [7]. Physiologically, in both species (i.e., Ochotona and Phenacomys), the ileal villi showed strong intestinal alkaline phosphatase (IAP) activity, an enzyme essentially linked with the absorption and homeostasis of the small intestine [8]. Interestingly, the villous epithelium of the caecal villi in Phenacomys showed an intense activity of IAP, whereas the caecal villi in Ochotona had a negative IAP expression, suggesting that the caecal villi of *Ochotona* may not serve the same function as the villi of the small intestine or of the ones of the caecal villi in *Phenacomys* [7].

The pika and other herbivores that possess a large caecum (e.g., rabbit, field vole, hyrax, beaver), rely on volatile fatty acids that are elaborated and absorbed in the caecum for metabolic energy. It seems that caecal digitations are specific structures that increase the absorption surface area for volatile fatty acids like ruminal papillae in ruminants [9].

A comparative morphological report of the caecal mucosa in pika and rabbits splits the caecal mucosa of both the rabbit and the pika into two portions, i.e. a higher portion (or the protruded region) and the lower portion (or the basal region) [9]. In rabbits, at 25 days of gestation, both regions of the caecal mucosa showed columnar villi. At 1 day after birth, caecal villi persisted in both regions but adopted a columnar appearance in the basal region and a round aspect in the folded region. At 5 days after birth, the protruded region still displayed round villi, but the fold of the caecal mucosa in this region grew higher in the next days forming the spiral fold, whereas, in the basal region, some regular short ridges were identified as derived from villi present at 1 day after birth. At 10 and 15 days after birth, both regions of the caecal mucosa showed only irregular gradually decreasing short ridges instead of previously existing villi, an aspect noticed in the adult period [9].

As a comparison, in pika, at 20 days of gestation, the protruded region of the caecal mucosa exhibited a rather straight fold while the basal zone consisted of columnar villi. At 25 days of gestation, the basal region exposed columnar villi whereas the fold of the protruded region showed a zigzag pattern and didn’t present villi [9]. After birth, on day one, the caecum grew wider, the basal region still maintaining the columnar villi, whereas the folds looked divided into several humplike protuberances that preserved the zigzag appearance. Shortly after (i.e., day 5 after birth), a significant decrease in the height of the villi in the basal region occurred, and the zigzagged humplike protuberances grew in height forming short and robust caecal digitations. On day 10 after birth, the basal region showed only irregular short ridges instead of the previously reported short villi, whereas the caecal digitations grew longer and did not display a zigzag pattern. Finally, on day 15 after birth, the caecal digitations grew longer adopting a long columnar shape, which eventually grew into slenderness flat on day 20 postpartum. The basal region appeared flatter as compared to the previous stage [9].

Caecal digitations, also called ”caecal villi” by some authors [7], appear to be specific structures in the caecum in pika, such digitations not being described in the large intestine in any other adult mammal. Some studies suggested that the slender caecal protrusions should be called caecal digitations [9] rather than villi for the reason that [1] caecal digitations are much larger than duodenal villi (e.g., 2–7/0.1–0.3 mm *vs* 0.5/0.07 mm); [2] villi can be detected all over the intestinal mucosa whereas the caecal digitations are restricted to the protruded region and are arranged in a single line; [3] the openings of the intestinal glands may occur on the surface of digitations, the pits not being present on the surface of villi [9]. The structures that should be named caecal villi are the ones existing during the late fetal period to 5 days after birth in pika and rabbits. Transitory villi were described in the large intestine of other mammals during the early suckling period, villi that eventually disappear. The specific purpose of the above-mentioned caecal villi during the suckling period is for a more efficient absorption of the maternal milk [10, 11].

Finally, domestic rabbit is a crucial multipurpose species by being considered an important meat livestock with great economic value, a valuable experimental animal, and, perhaps, a perfect pet. Recent experimental progress has shown that rabbits have many advantages by serving as a gap bridge between small animal models (suitable for discovery phases of research) and larger animal models frequently essential for translational (pre-clinical) research, with quite a number of peculiarities about the large intestinal wall [12], including in the morphology of the caecum [13]. Additionally, rabbits are phylogenetically nearer to primates than rodents which makes them a prime candidate model overall approximate to humans [14]. In immunology, rabbits represent the “production platform” for monoclonal/polyclonal antibodies and lately recombinant proteins [14]. In light of this, the new microanatomical features described in rabbits may perhaps bring essential details in understanding anatomical, physiological, and species-related peculiarities.

## Conclusions

This paper presents some unique features of the caecal mucosa in adult rabbits, namely caecal villi, structures infrequently noticed in other adult mammals in the large intestine. A microscopic and morphometric comparison of the regular jejunal villi vs. the newly described caecal villi was performed. The presence of caecal villi in adult rabbits emphasizes once more the major role of the caecum not only in fermentation but also in local absorption.

## Electronic supplementary material

Below is the link to the electronic supplementary material.

Supplementary Material 1: **Additional file 1 - Figure A1**. Distribution of enterocyte (E) and goblet cells (G) per 100 μm villous basal membrane of the jejunal vs caecal covering epithelium

## Data Availability

The datasets used and/or analysed during the current study are available from the corresponding author on reasonable request.

## Declarations

### Prior publication

Data have not been published previously.
