# Supplementary figures and images for: Caecal villi? A comparative histological and morphometric study of caecal and jejunal mucosa in adult rabbits
Source: Acta Vet Scand. 2024 Sep 12;66:50. doi: 10.1186/s13028-024-00770-w (PMC11396618; doi:10.1186/s13028-024-00770-w)

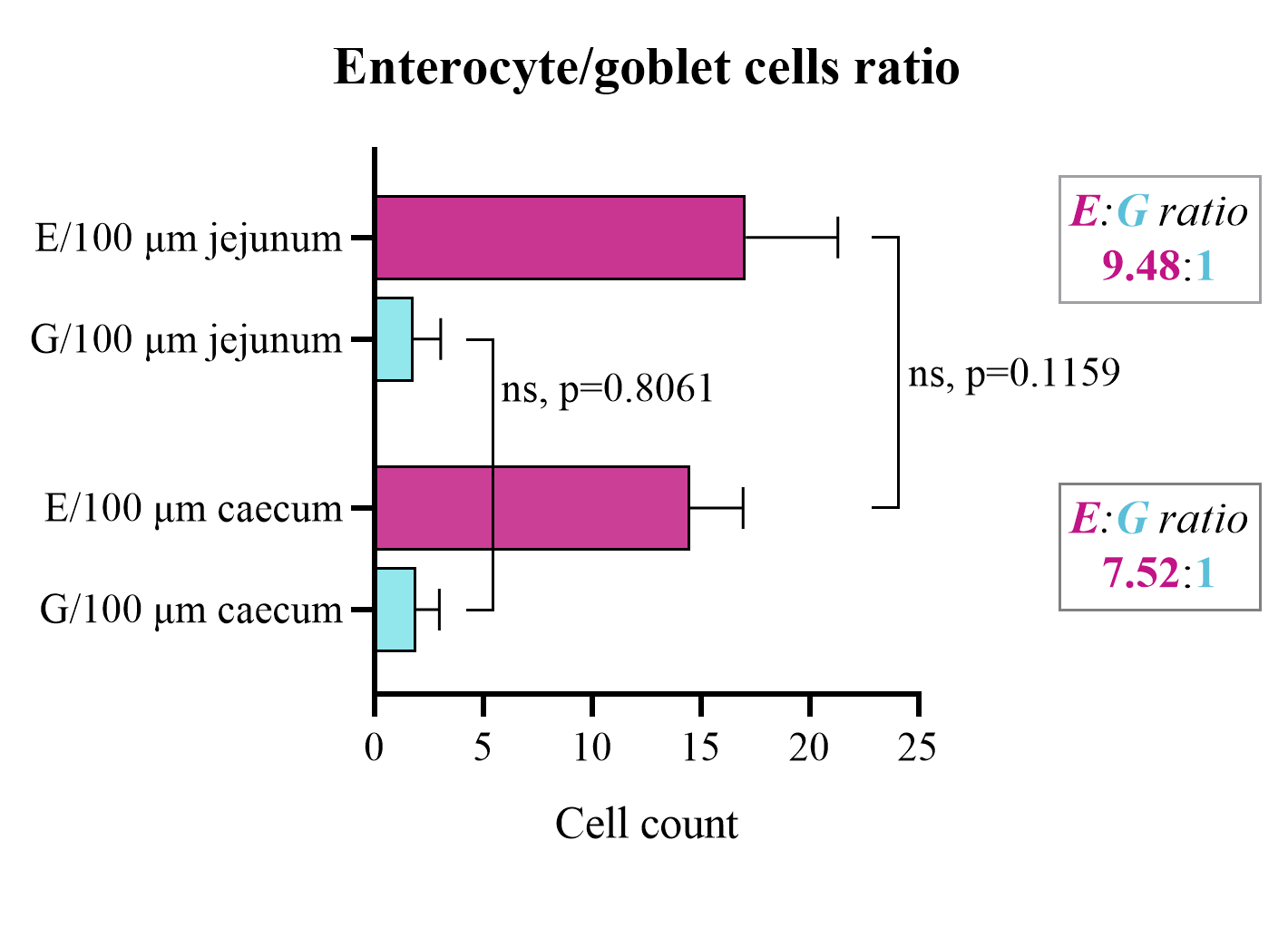

Supplement: Supplementary file 1 — Supplementary Material 1: Additional file 1 - Figure A1. Distribution of enterocyte (E) and goblet cells (G) per 100 μm villous basal membrane of the jejunal vs caecal covering epithelium [file 13028_2024_770_MOESM1_ESM.tif]
